# Economic evaluation of the prophylaxis for thromboembolism in critical care trial (E-PROTECT): study protocol for a randomized controlled trial

**DOI:** 10.1186/1745-6215-15-502

**Published:** 2014-12-20

**Authors:** Robert A Fowler, Nicole Mittmann, William H Geerts, Diane Heels-Ansdell, Michael K Gould, Gordon Guyatt, Murray Krahn, Simon Finfer, Ruxandra Pinto, Brian Chan, Orges Ormanidhi, Yaseen Arabi, Ismael Qushmaq, Marcelo G Rocha, Peter Dodek, Lauralyn McIntyre, Richard Hall, Niall D Ferguson, Sangeeta Mehta, John C Marshall, Christopher James Doig, John Muscedere, Michael J Jacka, James R Klinger, Nicholas Vlahakis, Neil Orford, Ian Seppelt, Yoanna K Skrobik, Sachin Sud, John F Cade, Jamie Cooper, Deborah Cook, On behalf of the Canadian Critical Care Trials Group and the Australia and New Zealand Intensive Care Society Clinical Trials Group

**Affiliations:** Sunnybrook Health Sciences Centre, University of Toronto, 2075 Bayview Avenue, Room D478, Toronto, ON M4N 3M5 Canada; Health Outcomes and PharmacoEconomic (HOPE) Research Centre, Sunnybrook Health Sciences Centre, Department of Pharmacology, University of Toronto, 2075 Bayview Avenue, E240, Toronto, ON M4N 3M5 Canada; Department of Medicine, Room D674, Sunnybrook Health Sciences Centre, Room D674, 2075 Bayview Avenue, Toronto, ON M4N 3M5 Canada; Department of Clinical Epidemiology & Biostatistics, Faculty of Health Sciences, McMaster University, 1280 Main Street West, HSC-2C12, Hamilton, ON L8S 4K1 Canada; Department of Research and Evaluation, Kaiser Permanente Southern California, 100 S Los Robles, Pasadena, CA 91101 USA; Department of Medicine, 144 College Street, Room 600, Toronto, ON M5S 3M2 Canada; The George Institute for Global Health, Royal North Shore Hospital, University of Sydney, Pacific Highway, St Leonards, NSW 2065 Australia; Institute of Health Policy, Management and Evaluation University of Toronto Health Sciences Building, 155 College Street, Suite 425, Toronto, ON M5T 3M6 Canada; Toronto Health Economics and Technology Assessment (THETA) Collaborative, Leslie Dan Pharmacy Building, University of Toronto, 144 College Street, 6th Floor, Toronto, ON M5S 3M2 Canada; Intensive Care Department, Medical Director, Respiratory Services, King Saud Bin Abdulaziz University for Health Sciences, King Abdulaziz Medical City, ICU 1425, PO Box 22490, Riyadh, 11426 Kingdom of Saudi Arabia; Department of Medicine, King Faisal Specialist Hospital & Research Centre-Gen. Org, PO Box 40047, Jeddah, 21499 MBC# J-46 Saudi Arabia; Department of Intensive Care, Hospitalar Santa Casa, Rua Professor Annes Dias, 295 - Centro Histórico, Porto Alegre, RS 90020-200 Brazil; Division of Critical Care Medicine, Center for Health Evaluation and Outcome Sciences, St Paul’s Hospital and University of British Columbia, 1081 Burrard St, Vancouver, BC V6Z 1Y6 Canada; Center for Health Evaluation and Outcome Sciences, 1081 Burrard Street, Vancouver, BC V6Z 1Y6 Canada; Department of Medicine (Critical Care), Ottawa Hospital, Ottawa Hospital Research Institute, Centre for Transfusion and Critical Care Research, 725 Parkdale Ave, Ottawa, ON K1Y 4E9 Canada; Departments of Anesthesiology, Medicine, Pharmacology and Surgery, Dalhousie University and the Capital District Health Authority, Halifax NS, Room 5452-Halifax Infirmary, 1796 Summer St, Halifax, NS B3H 3A7 Canada; Interdepartmental Division of Critical Care Medicine and Departments of Medicine & Physiology, University of Toronto, 600 University Avenue, Toronto, ON M5G 1X5 Canada; Department of Medicine, Division of Respirology, University Health Network and Mount Sinai Hospital, 600 University Avenue, Toronto, ON M5G 1X5 Canada; Department of Medicine and Interdepartmental Division of Critical Care, Mount Sinai Hospital and University of Toronto, 600 University Avenue, Toronto, ON M5G 1X5 Canada; Department of Surgery, Keenan Research Centre, Li Ka Shing Knowledge Institute, St Michael’s Hospital, University of Toronto, 4-007 Bond Wing, St Michael’s Hospital, 30 Bond Street, Toronto, M5B 1W8 Canada; Department of Community Health Sciences, Departments of Critical Care Medicine, Attending Physician, Foothills Medical Centre Multisystem Intensive Care Unit, Alberta Health Services, University of Calgary, Room 3D39, Teaching Research and Wellness Building, 3280 Hospital Dr NW, Calgary, AB T2N 4Z6 Canada; Department of Medicine, Angada 4 Kingston General Hospital, 76 Stuart Street, Kingston, ON K7L 2V7 Canada; Department of Anesthesiology and Critical Care, University of Alberta Hospital, 8440-112 St, Edmonton, AB T6G 2B7 Canada; Division of Pulmonary, Sleep and Critical Care Medicine, Rhode Island Hospital, Professor of Medicine, Alpert Medical School of Brown University, 222 Richmond Street, Providence, RI 02903 USA; Department of Pulmonary & Critical Care Medicine, Mayo Clinic, 200 First Street, SW, Rochester, MN 55905 USA; Intensive Care Barwon Health, Australian and New Zealand Intensive Care Research Centre, Monash University School of Medicine, 99 Commercial Road, Geelong, VIC 3004 Australia; Intensive Care Barwon Health, Australian and New Zealand Intensive Care Research Centre, Deakin University, 1 Gheringhap Street, Geelong, VIC 3220 Australia; Critical Care Medicine, Nepean Hospital, Derby Street, Penrith, NSW 2747 Australia; Critical Care Medicine, Hôpital Maisonneuve-Rosemont, 5415 Blvd. De l’Assomption, Montreal, QC H1T 2M4 Canada; Department of Medicine, University Trillium Hospital, 100 Queensway West, Toronto, ON L5B 1B8 Canada; Intensive Care Unit, Royal Melbourne Hospital, Grattan Street, Parkville, VIC 3050 Australia; ANZIC-RC Department of Epidemiology and Preventive Medicine Monash University, The Alfred Centre Level 6, 99 Commercial Road, Melbourne, VIC 3004 Australia; Departments of Medicine, Clinical Epidemiology & Biostatistics, McMaster University, 1280 Main Street West, Hamilton, ON L8S 4K1 Canada

**Keywords:** Economic, Cost-effectiveness, Venous, Thromboembolism, PROTECT, Unfractionated, Heparin, Low molecular weight, Intensive, Critical

## Abstract

**Background:**

Venous thromboembolism (VTE) is a common complication of critical illness with important clinical consequences. The Prophylaxis for ThromboEmbolism in Critical Care Trial (PROTECT) is a multicenter, blinded, randomized controlled trial comparing the effectiveness of the two most common pharmocoprevention strategies, unfractionated heparin (UFH) and low molecular weight heparin (LMWH) dalteparin, in medical-surgical patients in the intensive care unit (ICU). E-PROTECT is a prospective and concurrent economic evaluation of the PROTECT trial.

**Methods/Design:**

The primary objective of E-PROTECT is to identify and quantify the total (direct and indirect, variable and fixed) costs associated with the management of critically ill patients participating in the PROTECT trial, and, to combine costs and outcome results to determine the incremental cost-effectiveness of LMWH versus UFH, from the acute healthcare system perspective, over a data-rich time horizon of ICU admission and hospital admission. We derive baseline characteristics and probabilities of in-ICU and in-hospital events from all enrolled patients. Total costs are derived from centers, proportional to the numbers of patients enrolled in each country. Direct costs include medication, physician and other personnel costs, diagnostic radiology and laboratory testing, operative and non-operative procedures, costs associated with bleeding, transfusions and treatment-related complications. Indirect costs include ICU and hospital ward overhead costs. Outcomes are the ratio of incremental costs per incremental effects of LMWH versus UFH during hospitalization; incremental cost to prevent a thrombosis at any site (primary outcome); incremental cost to prevent a pulmonary embolism, deep vein thrombosis, major bleeding event or episode of heparin-induced thrombocytopenia (secondary outcomes) and incremental cost per life-year gained (tertiary outcome). Pre-specified subgroups and sensitivity analyses will be performed and confidence intervals for the estimates of incremental cost-effectiveness will be obtained using bootstrapping.

**Discussion:**

This economic evaluation employs a prospective costing methodology concurrent with a randomized controlled blinded clinical trial, with a pre-specified analytic plan, outcome measures, subgroup and sensitivity analyses. This economic evaluation has received only peer-reviewed funding and funders will not play a role in the generation, analysis or decision to submit the manuscripts for publication.

**Trial registration:**

Clinicaltrials.gov Identifier: NCT00182143. Date of registration: 10 September 2005.

**Electronic supplementary material:**

The online version of this article (doi:10.1186/1745-6215-15-502) contains supplementary material, which is available to authorized users.

## Background

Venous thromboembolism (VTE) is a common complication of critical illness and has important clinical consequences, including deep vein thrombosis (DVT), pulmonary embolism (PE), increased length of stay in the intensive care unit (ICU) and hospital and death [1–3]. Although DVT has potentially serious consequences, it is often unrecognized in the ICU as the clinical examination for DVT lacks sensitivity and specificity [4]. Further, routine ultrasound screening for DVT is not a cost-effective diagnostic strategy in practice [5]. Thus, thromboprophylaxis is the most appropriate mechanism to prevent VTE and its complications among critically ill patients.

We have previously documented that most economic evaluations of VTE strategies are designed after the results of the trial are known, and are funded by the manufacturers of the agents compared [6]. This introduces the opportunity for biased design and interpretation of economic evaluations. Therefore, we designed the economic evaluation (E-PROTECT) of a multicenter randomized, blinded controlled trial of 3,746 patients comparing the effectiveness of unfractionated heparin (UFH) and low molecular weight heparin (LMWH) (dalteparin) [7]. The primary objective of E-PROTECT is to identify and quantify the total (direct and indirect, variable and fixed) costs associated with the management of critically ill patients participating in the PROTECT trial, and to combine costs and outcome results to determine the incremental cost-effectiveness of LMWH versus UFH, from the acute healthcare system perspective, over a data-rich time horizon of the ICU admission and hospital admission.

## Methods/Design

### E-PROTECT background studies and methodologies

#### The PROTECT trial

The objective of the Canadian Institutes of Health Research (CIHR)-funded PROTECT trial [7, 8] was to evaluate LMWH versus UFH on the primary outcome of the incidence of proximal leg DVT diagnosed by compression ultrasound, with secondary outcomes of PE, venous thrombosis at any site, bleeding and heparin-induced thrombocytopenia (HIT). The study design was a randomized, stratified, concealed double-blind multicenter trial with enrolment throughout Canada, Australia, the United States, Saudi Arabia, the United Kingdom and Brazil. The PROTECT trial enrolled 3,746 critically ill patients between May 2006 and June 2010 according to previously published eligibility criteria [7, 8]. A central computerized web-based or phone-in randomization system ensured concealed randomization of patients to either dalteparin 5,000 international units (IU) daily or UFH 5,000 IU twice daily subcutaneously for the ICU stay. Only the research pharmacist at each participating centre was aware of the allocation. Bilateral proximal leg compression ultrasounds were performed within 48 hours of ICU admission, twice weekly and on DVT suspicion. A predefined algorithm was used to diagnose PE; bleeding, HIT, other thrombosis and complications were identified using *a priori* definitions and procedures. The PROTECT publication itself provides complete study data [7].

#### The E-PROTECT pilot study

To first determine the feasibility of obtaining patient-specific line-item costing (for each aspect of care delivery), we conducted a pilot study between 2006 and 2007 involving six hospitals in Canada, the United States and Australia [9]. However, we discovered that in both privately funded and publically funded institutions, the variability around patient costing was substantial and that line-item costs were not routinely available. Many costs were ‘rolled up’ into summary cost measures, and subsequently, this methodology would not allow for a linkage of costs and clinical events to be measured as part of the PROTECT trial case report form. Therefore, we designed a more appropriate cost gathering methodology to capture hospital-specific line-item costs according to important variables that we anticipated will drive costs and possible cost-effectiveness (Additional file 1).

In order to determine such cost drivers, we performed a systematic review of economic analyses of thromboprophylaxis strategies in hospitalized patients to identify variables that we anticipate will drive costs and possible cost-effectiveness in E-PROTECT, and to determine potential ranges for willingness-to-pay to avoid DVT and PE [6]. From 5,180 potentially relevant studies, 39 met the eligibility criteria from which we extracted data on study characteristics, quality, costs and efficacy. In addition to identifying variables likely to be influential in E-PROTECT, we found that LMWHs appear to be the most economically attractive drugs for VTE prevention in acutely ill hospitalized patients, whereas newer agents may be more economically attractive in patients receiving joint replacement surgeries. However, the manufacturer of the new agent supported approximately two-thirds of evaluations and such drugs were likely to be reported as economically favorable. Incremental cost-effectiveness ratios to prevent VTE events ranged from a dominance of LMWH to under $5,000 per VTE event avoided [6].

### E-PROTECT methods

#### E-PROTECT design and economic assumptions

We designed E-PROTECT before the results of the PROTECT trial were known. Study funding was from peer-reviewed sources and none of the funders played a role in the generation, analysis or decision to submit the economic evaluation for publication. We developed our analysis according to established guidelines [10–14]. Also, we used an acute healthcare system perspective (during the period of hospitalization) to encompass all in-patient direct medical and hospital costs, including physician and other personnel costs. Our preliminary analytic plan was pre-specified with public study funders (Heart and Stroke Foundation, Ontario, Canada) as part of the economic evaluation of the PROTECT trial protocol prior to completion of the trial and unblinding of the economic consequences of clinical data.

#### E-PROTECT patients, outcomes and effects

No patients were lost to follow-up. Although the PROTECT trial considered both intention-to-treat and on-treatment analyses, primary outcomes were based on adherence to the intention-to-treat principle and will form the clinical event estimates of the economic analyses. We recorded the frequency of DVT, PE, major bleeding and HIT among all patients to measure the primary clinical incremental effect of the difference in any VTE (all limb DVT, PE and non-limb thromboses), and secondary clinical incremental effects of episodes of PE, DVT, major bleeding and HIT. The PROTECT trial was designed and powered to evaluate differences in the rate of thrombotic events between two pharmacologic thromboprophylaxis strategies, not differences in life expectancy; accordingly, incremental differences in life years are not primary outcomes in this economic evaluation. All measures of clinical events, medications administered, laboratory and radiology testing, complications, blood product transfusions, procedures and surgeries and duration of ventilation, ICU and hospital stay were recorded alongside the PROTECT trial as part of the study case report form [7, 8].

### E-PROTECT costs

#### Total, direct and indirect costs

Total costs for patients in the PROTECT trial comprise direct and indirect costs [15]. To determine which cost to include, we performed a systematic review of the VTE cost-effectiveness literature [6] for hospitalized patients, and reviewed evidence underlying the relative importance among the E-PROTECT Steering Committee, before deciding upon final cost variables. Direct costs are attributable to medications, transfusions, laboratory or radiology tests or procedures or personnel directly associated with specific components of patient care. Such costs can usually be determined on an item-by-item, procedure-by-procedure basis. Indirect costs are those for services or procedures that often benefit more than one patient at a time and their precise benefits to specific patients are often difficult to trace, such as maintenance of equipment and infrastructure. A large proportion of such indirect costs comprise the ‘overhead’ in operating an ICU, laboratory or pharmacy, for example [10, 15, 16]. These costs can be fixed (roughly the same amount on a recurring basis), or variable (fluctuating depending upon patient circumstances and course). E-PROTECT measured both direct and estimated indirect costs at study sites.

#### Direct costs from participating sites

Direct and indirect costs were sought from 23 of 67 hospitals and five of six countries participating in the PROTECT trial (12 hospitals in Canada; five hospitals in Australia; three hospitals in the United States; two hospitals in Saudi Arabia; one hospital in Brazil). Sites will be invited to participate in the costing component of the economic evaluation to reflect overall proportions of patients enrolled in the PROTECT trial among all participating countries. Direct costs will be divided among the following categories: 1) study-related drugs (unfractionated heparin, dalteparin, enoxaparin, tinzaparin, other low molecular weight heparins, protamine, Desmopressin (DDAVP), aprotinin, aminocaproic acid, danaparoid, lepirudin, argatroban, fondaparinux, activated Factor VII, acetyl salicylic acid (ASA), clopidogrel, a representative proton-pump inhibitor (pantoprazole), vitamin K, warfarin and epinephrine for injection or infusion); 2) laboratory testing (complete blood count, electrolytes, creatinine, blood urea nitrogen, arterial blood gas, partial thromboplastin and prothrombin time, anti-Xa level, enzyme-linked immunosorbent assay (ELISA) and serotonin release HIT assay); 3) personnel (including *per diem* most responsible physician and per consultation physician charges over the course of an ICU and hospital admission, nursing, pharmacist, respiratory therapist, physical therapist, social work and ICU administrative and/or clerical staffing hourly wage range); 4) radiology (unilateral and bilateral leg compression ultrasonography and venography, portable chest radiograph, computerized tomography (CT) scan of the chest with an angiogram (pulmonary embolism protocol), CT scan of the abdomen, pelvis, and head, pulmonary angiogram and ventilation-perfusion scan); 5) procedural costs (electrocardiogram, central venous catheter material costs, inferior vena cava filter material and insertion and removal costs, naso- or oro-gastric tube, procedure costs for gastroscopy, colonoscopy, bronchoscopy, and angiography with embolization and intermittent and continuous renal replacement therapy); and, 6) operative costs for laparotomy (including the surgeon and surgical assistant, anesthesiology and nursing personnel costs).

Operative and bedside surgical procedures were classified according to procedure and body site (head and neck, cardiac, vascular, thoracic, gastrointestinal, orthopedic, genitourinary, plastic, neurosurgical or other), by two independent adjudicators, blinded to treatment allocation, with separate attribution as to whether the procedure was conceivably precipitated or related to the study drug (bleeding or thromboses). Disagreements were resolved by consensus or a third reviewer. Rates of all surgeries and study drug-related surgeries will be compared for each study group and those we believe to be possibly related to the study drug will be included in the cost analysis.

#### Direct costs associated with heparin-induced thrombocytopenia

As episodes of HIT with thrombosis (HITT) and without thrombosis involve complex and expensive investigation and treatment, some authors previously performed a single-center micro-costing study in one PROTECT trial center. They aimed to estimate the total average attributable costs of an episode of HIT (and HITT including arterial and venous thromboses), as well as associated HIT-safe anticoagulants, associated bleeding and specialized HIT diagnostic testing. The details of the micro-costing study are published elsewhere [17] but included diagnosis, investigation, treatment and administration costs. However, for E-PROTECT, we will capture costs and measures of resource use for complications such as bleeding, HIT and HITT as part of the PROTECT case report and centre-specific E-PROTECT costs, and will not assign additional HIT or bleeding-specific costs in order to avoid double counting. Bleeding and blood product transfusion services costs (per unit of red blood cells, frozen plasma and thawing, cryoprecipitate, cross matching and platelets and associated laboratory testing of blood type and screen) were derived from a Canadian national transfusion resource, education and costing exercise [18].

#### Prophylactic and therapeutic anticoagulation outside the ICU

Ward-based prophylactic and therapeutic anticoagulation was not directly measured; therefore, we will make the following assumptions:

1. If no therapeutic anticoagulation was used in last two days in ICU, we will assume UFH prophylaxis will be used for the entire ward stay;
2. If warfarin was used in last two days in ICU, we will assume that warfarin will be used for entire ward stay;
3. If treatment doses of LMWH were used in last two days in ICU, we will assume that treatment with LMWH will continue for five days on the ward, followed by warfarin for the remainder of the hospital stay;
4. If treatment doses of UFH were used in last two days in ICU, we will assume that treatment with UFH occurs for five days on the ward, followed by warfarin for the remainder of the hospital stay;
5. If treatment doses of danaparoid or other HIT-safe anticoagulants were used in last two days in ICU, we will assume that treatment with danaparoid or other HIT-safe anticoagulants occurs for five days on the ward, followed by warfarin for the remainder of the hospital stay.

#### Indirect costs - ICU and ward care

We will measure institution-specific estimates of indirect (overhead) costs per day of care in the center’s highest acuity ICU (capable of providing ventilation and hemodynamic support), and for patients receiving care on standard medical and surgical wards. From the E-PROTECT pilot, we found that each institution determined this cost per day in a unique fashion, but generally comprised components of non-physician personnel cost, equipment maintenance, some diagnostic testing and ICU and other ward overhead infrastructure costs [9]. This rolled-up indirect per day cost will be determined to estimate the difference between the total of individual direct costs relevant for patients in the PROTECT trial (above), and the total daily costs of care that may reflect additional and indirect costs which are important in determining total costs, but are difficult to capture as single variables.

Mean institutional costs of ICU care typically represent very high costs on the first day, and substantially lower (one-quarter) costs after the second day [16], with a further decrease after discharge to a ward bed. Using our hospital estimates of indirect costs in ICUs and on the ward, and in accordance with prior literature, we will apply a ratio of 1 (hospital ward):2.5 (ICU day two onwards):4 (ICU day one) [16, 19–21]. Most daily costs of care in ICUs are fixed, not variable, and prior work has demonstrated minimal direct-variable cost differences due to ventilation status [16]. Because of this and because the vast majority of ICU days represent days patients received mechanical ventilation, we will apply a small incremental (between 3 and 5%) daily indirect cost due to this advanced life support [16].

#### Direct and indirect cost data collection

We measured all costs at each site using a costing operations manual outlining a standardized multistep process (Additional file 1). Between 2007 and 2011, the principal investigator and a research assistant contacted the site principal investigator and research coordinator at each participating center with the list of variable costs required, who then subsequently contacted the most appropriate individual in each hospital’s accounting, human resources, pharmacy, radiology and laboratory services and blood bank. Standard definitions (per unit of measurement or time, and so on) were created and explained and hospital-specific costs requested for each variable. Where costs were influenced by extra-hospital jurisdictions (such as jurisdictional payer physician costs, unionized employees and reference laboratory costs), these were recorded. In all cases, costs were requested but if only charges were known, then we converted to costs by the institution’s cost-to-charge estimate for that item. Both professional (performance and/or interpretation) and technical costs were recorded for procedures when applicable. We undertook an iterative series of communications until no further data could be gleaned. All data are without patient identifiers and are be maintained in password-protected and encrypted laptop or desktop, in locked offices.

#### Exploring cost variability among sites

To explore variability among centers and countries, cost data will be summarized by means (and standard deviations) and medians (and interquartile ranges) among all sites, and by country for all variables, as per our pilot costing study [9]. Visible outliers will be reconfirmed with individual hospital contacts before determining median costs among all centers for each variable in the dataset to be used for all primary and secondary analyses. Data from sites that have cost accounting methodologies precluding the inclusion of certain data will be noted, but multi-country median costs will be used to improve the generalizability of our estimates and to mitigate the influence of high and low cost outliers. Prior to final analyses being undertaken, participating sites will be re-queried to determine if particular costs have changed substantially (for example, by more than 25%), beyond inflationary or deflationary changes, over the course of the study and if so, the most current costs will be used. Country and year-specific costs will be converted to US dollars, first accounting for annual inflation [22–25]. We plan on using international currency conversion, instead of purchase power parity (PPP)-based conversions, because health-specific PPPs are not available for all participating countries, and non-health PPP conversion rates vary substantially over the period of the analysis [23]. Country-specific costs will be considered only in sensitivity analyses. As of 1 June 2013, 1 United States dollar was worth approximately 1.03 Canadian dollars, 1.04 Australian dollars, 2.12 Brazilian Real, and 3.75 Saudi Arabian Riyal [23].

### E-PROTECT analytic plan

The base case cost-efficacy ratio is the ratio of incremental costs per incremental effects of LMWH versus unfractionated heparin during the period of hospitalization. Incremental costs will be derived for all patients by first calculating total costs for all patients in each arm of the PROTECT trial. Item costs will be multiplied by the frequency or event rates for medications administered, laboratory and radiological tests incurred, other diagnostic or therapeutic procedures performed, transfusions received, per day personnel and ICU or ward costs, depending upon number of events and length of stay in the ICU and hospital. Total, then median and mean per patient costs for each of the LMWH and unfractionated heparin groups will be calculated. Incremental costs will be taken as the difference in median per patient costs between groups; however, mean costs will also be provided in order to generate total costs. Effects for the base case analysis will be calculated separately for each primary (all thromboses), secondary (PE, DVT, major bleeding and HIT) and tertiary (survival) endpoints. Incremental effects will be taken as the difference in per patient event rates between groups. Therefore cost-effectiveness ratios will be displayed in terms of incremental costs divided by incremental effects, specifically, the cost to prevent a thrombosis at any site, the cost to prevent a PE, DVT, major bleeding event and episode of HIT, and life-year gained as is commonly used in cost-effectiveness analysis for VTE prevention strategies (Box 2) [6]. If costs are less in the LMWH group, a cost-minimization analysis will be reported, highlighting the difference in costs (with 95% confidence intervals) between groups.

### Evaluation Framework Overview

1. **Question**: Is the use of LMWH as compared to UFH cost-effective for the prevention of VTE in critically ill medical-surgical patients?
2. **Perspective**: Our primary perspective is the acute healthcare system perspective. We will perform sensitivity analyses from various geographic regions participating in the PROTECT trial.
3. **Clinical outcomes**: Rates of all thromboses, DVT, PE and complications (for example, bleeding and HIT), length of stay and mortality (ICU and hospital).
4. **Costs**: All direct and indirect medical costs associated with treatment and complications (such as ICU and non-ICU hospitalization, personnel, medications, diagnostic testing and procedures) will be identified and evaluated. Costs will be converted to Canadian and United States (2013 dollar rates) costs.
5. **Evaluation**: Our primary outcome is the incremental cost per in-hospital thrombotic event avoided. Long-term results will be presented in terms of costs per life year and quality-adjusted life years gained, using utilities from studies of appropriate duration, to allow for broad comparison of cost-efficacy with other medical interventions.
6. **Comparators**: We will compare LMWH with UFH for outcomes and associated costs.
7. **Time horizon**: We will focus on near-term horizons; costs and incremental cost effectiveness ratio outcomes at ICU and hospital discharge. We will model long-term outcomes over a lifetime horizon.
8. **Discounting**: We will discount cost and utilities at 3% for any longer-term (over one year) analyses.
9. **Uncertainty**: We will use sensitivity analyses and bootstrapping to produce confidence intervals.

DVT = deep vein thrombosis; HIT = heparin induced thrombocytopenia; ICER = incremental cost-effectiveness ratio; ICU = intensive care unit; LMWH = low molecular weight heparin; PE = pulmonary embolism; UFH = unfractionated heparin; VTE = venous thromboembolism.

### Outcomes Overview

1. **Primary outcome**:
  Incremental cost per thromboses (any site) avoided
2. **Secondary outcomes**:
  (i.) Incremental cost per PE avoided
  (ii.) Incremental cost per DVT avoided
  (iii.) Incremental cost per major bleeding event avoided
  (iv.) Incremental cost per episode of HIT avoided
3. **Tertiary outcomes**:
4. Incremental cost per life year and quality-adjusted life year gained (lifetime horizon)

### E-PROTECT subgroups

As subgroup analyses, we will investigate specific patients in the PROTECT trial who may have differential effects and costs as compared to the entire population, including patients with higher and lower severity of illness (APACHE^1^ II score ≥25 versus <25), patients with a body mass index of ≥40 kg/m^2^ versus <40 kg/m^2^, patients requiring inotropes or vasopressors at enrollment versus those who do not and medical versus surgical patients. Although ventilation status was initially planned as a subgroup, approximately 90% of patients were mechanically ventilated at enrollment [7] and thus analysis in this subgroup will not be informative. We will directly calculate the incremental cost difference and generate 95% confidence intervals using the bias corrected and accelerated (BCa) method in R, among 1,000 bootstrap samples. We will examine the relative influence of all individual costs using a Tornado diagram.

### E-PROTECT sensitivity analyses

As patient characteristics, effects and costs may differ outside of tightly controlled clinical trials and in various jurisdictions, we plan a number of sensitivity analyses to explore how incremental cost-effectiveness ratios may change with plausible differences in values, including costs of LMWH and UFH, and per day cost of care in ICU and hospital wards. We will also perform sensitivity analyses using the efficacy analysis of the PROTECT trial that considered only patients who received the study drug for two days or more and had two or more leg compression ultrasounds, and by considering only symptomatic thromboses. Multi-way sensitivity analyses will be performed by varying the estimates of pairs of potentially influential variables. We will perform a probabilistic sensitivity analysis of pairs of known costs and effects from the PROTECT trial, using bootstrapping techniques, among 1,000 samples, with replacement, in order to generate an incremental cost-effectiveness plot [26, 27].

### Secondary analyses

#### E-PROTECT post-hospital discharge modeling

**Figure 1:**
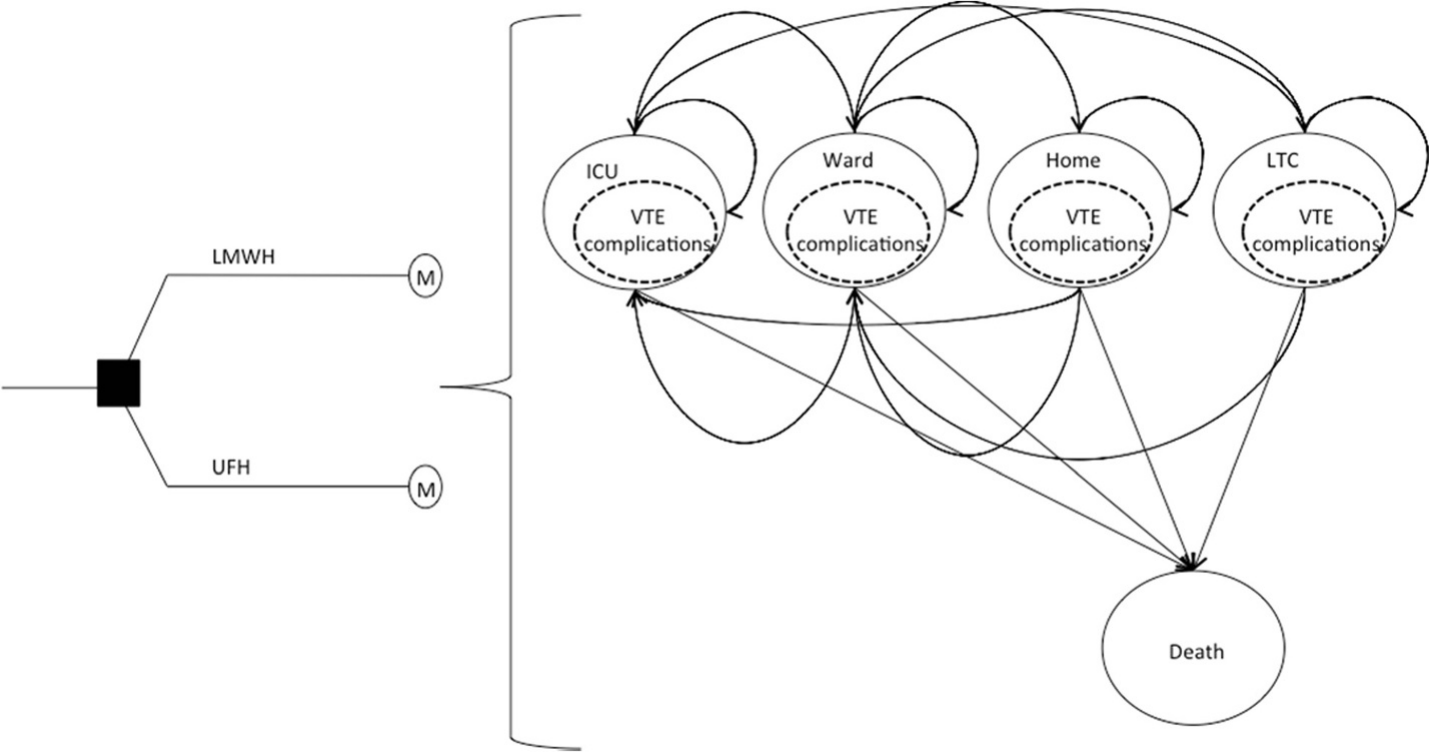
**Markov model for long**-**term outcomes and costs of critically ill patients in E-** **PROTECT.** See Sud *et al*. [5]. ICU = intensive care unit; LMWH = low molecular weight heparin; LTC = long-term care; M = Markov node; UFH = unfractionated heparin; VTE = venous thromboembolism.

Some clinical events may occur after ICU and hospital discharge and these events may utilize resources (such as re-admission to the ICU, rehabilitation and physician visits). Because we cannot follow all patients in the PROTECT trial over their actual life expectancy, we may use discharge vital statistics and known post-critical illness follow-up literature to model the lifetime effects and cost-efficacy ratios of prevention strategies for all patients in the PROTECT trial, depending upon the results for the in-hospital time horizon. We will know clinical events and outcomes until death or discharge from hospital. However, if the primary analysis implies that long-term effects and costs are important to explore (particularly if the primary analysis demonstrates one strategy is more costly but with greater effects), post-discharge, a Markov-based process (Figure 1) will be used to model longer-term events and will allow patients to transition between states of health or illness until all patients in the model have died [5].

Both background mortality and increased mortality due to the initial illness, thrombosis event or complication will be considered. The cycle length will be one month. Transition probabilities will be taken from the E-PROTECT follow-up cohort and literature of post-ICU survival. The E-PROTECT long-term model is informed by the structures of published models for prophylaxis with LMWH in other populations [5] and the actual course of the patients in the PROTECT trial. We will combine these data, and known utilities of post-ICU discharge states, with outcomes and life years determined by the PROTECT trial and the long-term model in order to derive a measure of quality-adjusted life years and associated incremental cost-efficacy ratios. Given our in-hospital primary time horizon and focus on thrombotic events in the PROTECT trial, we intentionally will not directly measure short-term health-related quality of life measurements for our primary time horizon, but will apply measures of health-related quality of life for modeled post-hospital survival using data from longitudinal studies of critically ill patients over an appropriate time horizon [28–36]. We will apply 3% discounting for all effects and costs for any lifetime modeled events.

### Statistical analyses

All primary analyses will be based on the intention-to-treat principle, as per the primary analyses for the PROTECT trial. We use descriptive analyses to describe the baseline characteristics. Means (and standard deviations) or medians (and interquartile ranges) will be used to describe average effect and cost estimates, and Chi-square tests and two-sample t-test comparisons will be used as appropriate. We will test for differences in costs using standard non-parametric tests, and use nonparametric analysis of variance techniques to test for interactions with the heparin strategies. For missing or unavailable data, we will use imputation of the median value from the appropriate subgroups in E-PROTECT and compare results obtained with and without imputation to ensure large variations do not exist [37, 38]. Statistical significance for differences among *a priori* comparisons will be set at a P value of 0.05. Primary analysis will be undertaken using Excel (Microsoft Corp, Redmond Washington, United States), R (Free Software Foundation) and SAS (Cary, North Carolina, United States). Post-hospital discharge models will be constructed using Excel and TreeAge software (TreeAge Software Inc., Williamstown, Massachusetts, United States).

### Ethics

Research ethics approval for E-PROTECT was granted by Sunnybrook Health Sciences Centre (project identifier: 115-2007). All other sites participating in the economic evaluation of the PROTECT trial have obtained research ethics approval for involvement in the PROTECT trial, or appropriate approval to include non-specific patient-based costing data (Canada: Queen Elizabeth II Hospital (Halifax); Hôpital Maisonneuve-Rosemont (Montréal); Ottawa Hospital (Ottawa); Kingston General Hospital (Kingston); Mount Sinai Hospital, University Health Network, St Michael’s Hospital, Sunnybrook Health Sciences Centre (Toronto); St Joseph’s Hospital (Hamilton); Foothills Hospital (Calgary); University of Alberta Hospital (Edmonton); St Paul’s Hospital (Vancouver). Australia: Royal North Shore Hospital (Sydney); Barwon Health (Geelong); Nepean Hospital (Penrith); Royal Melbourne Hospital, The Alfred Centre (Melbourne). United States: Rhode Island Hospital (Providence); Mayo Clinic (Rochester). Saudi Arabia: King Saud Bin Abdulaziz University for Health Sciences (Riyadh); King Faisal Specialist Hospital & Research Centre (Jeddah). Brazil: Hospitalar Santa Casa (Porto Alegre).

Informed consent was obtained from each participant in the PROTECT trial, or their substitute decision-maker, in accordance with local research ethics approvals.

### Study oversight

Study operations, methods, submission for funding and manuscript generation are under the oversight of the E-PROTECT steering committee (RF, NM, DC, WG, MG, GG and MK) on behalf of the Canadian Critical Care Trials Group.

## Discussion

The PROTECT trial is the largest trial undertaken of VTE prophylaxis for critically ill patients. Although LMWH has been shown to be an effective means of VTE prophylaxis for many patient populations, it has historically been associated with a higher drug acquisition cost than UFH, and the relative effects, side-effects and broader costs of VTE prophylaxis are unknown. A prospective economic evaluation is critical to the interpretation of the results of the PROTECT trial. The PROTECT trial results suggest that LMWH may reduce the frequency of pulmonary embolus and heparin-induced thrombocytopenia. Thus, physicians, pharmacists and policy makers will need to know whether the cost provides good value for the healthcare dollar. An economic evaluation is still critical for alternative clinical outcomes, particularly for differences in the adverse event profile between treatments that may lead to additional resource consumption. E-PROTECT provides an opportunity to answer these questions and address the cost-efficacy of VTE prevention using results with minimal risk of bias.

In preparation for E-PROTECT, we have performed a number of preliminary and sub-studies, including a systematic review of the VTE economic evaluation literature to inform cost-drivers, the E-PROTECT pilot study in three countries to determine feasible and valid cost data collection, and we have developed a decision-analytic model to explore both the importance of protocoled screening versus clinical case finding for VTE, and evaluate the long-term outcomes of various detection and treatment strategies [5, 6, 9].

Prospective measurement of economic and health outcome data alongside randomized controlled trials, although previously rare, has become increasingly common. There are several advantages [10]. Firstly, the costs of collecting clinical, economic and long-term follow-up data can be reduced if they are collected simultaneously. Secondly, certain data, such as information resource use, are impractical or impossible to gather retrospectively. Thirdly, the analysis can take full advantage of randomization to collect data unlikely to be confounded by differences in baseline characteristics between treatment arms. Fourthly, all elements of the analysis, including decisions regarding modeling assumptions and sensitivity analysis parameters and ranges, can be specified before investigators see unblinded data, reducing the risk of investigator bias. Finally, the economic analysis results can be generated at, or only slightly after, the time that clinical results are available. Such timely economic data can be particularly useful to those making budgetary and healthcare resource allocation decisions, especially if the intervention that is being evaluated is already in current practice. By conducting our economic analysis concurrent with the PROTECT trial, we take advantage of each of these strengths.

The major disadvantage of conducting an economic analysis concurrently with a randomized clinical trial is that a randomized clinical trial may not represent the same effects and costs as routine clinical practice. However, both the PROTECT and E-PROTECT trials address this limitation in that we will expose estimates generated from the PROTECT trial to adjustments in medical and surgical case-mix and severity of illness, intention-to-treat and on-treatment estimates, among others. Another potential disadvantage is that the primary outcome of the PROTECT trial was proximal leg DVT during ICU admission, confirmed by ultrasound, but which in critically ill patients is usually asymptomatic. However, the other venous thrombosis outcomes of the DVT and PE arm were largely clinically suspected and also objectively confirmed.

There are other aspects of the E-PROTECT trial methodology that deserve consideration. Firstly, our primary perspective is from the acute healthcare system (not societal) and our primary time horizon is in-hospital, not lifetime. This is because patients in the ICU receiving thromboprophylaxis do not receive it beyond their hospital stay; the intervention is focused on the reduction of in-hospital VTE events and thus the E-PROTECT trial is focused upon in-hospital costs. However, we will estimate post-discharge events and costs using our previously developed models, should the primary analyses indicate this to be informative [5].

Secondly, the primary outcome of E-PROTECT is the incremental cost to avoid a VTE event, not the incremental cost per life-year gained. This is because the PROTECT trial is not designed to estimate differences in survival, nor powered to detect differences in life expectancy; thus, it would be inappropriate to make this a primary outcome for E-PROTECT. However, if there were differences in life expectancy during the PROTECT trial we would address this as a tertiary outcome. Whereas there are broadly discussed thresholds of willingness-to-pay for a year of quality-adjusted life, there are no clear cost thresholds for VTE event avoidance. To address this, in our systematic review of economic evaluations in the VTE literature, we have summarized the ranges of incremental costs associated with various pharmacoprevention strategies. However, if E-PROTECT determines an incremental cost of LMWH over UFH, we will perform a specific follow-up study for E-PROTECT that will seek to establish such a threshold among patients, physicians, pharmacists and policy makers.

Thirdly, we capture both direct and indirect costs in E-PROTECT by seeking acute healthcare system line-item costs as opposed to line-item costs of each patient enrolled in the PROTECT trial. This stems from our pilot study demonstrating widely variable institution practices in reporting patient-specific cost accounting. We will derive valid direct and indirect costs of care, and avoid double counting among individual resources used and institution specific rolled-up patient and overhead costs.

Fourthly, we have chosen to gather costs from acute healthcare systems in many countries participating in the PROTECT trial. Although this has the potential to introduce variability in cost estimates, we believe this will help our findings be generalizable to more than one system. We will use international currency conversion instead of PPP conversions, because health-specific PPPs are not available for Saudi Arabia, and non-health PPP conversion rates vary substantially over the period of the analysis [23]. We will report our findings in a common currency, using appropriate currency conversion techniques, and adjusted for country specific inflation and deflation from the date of data acquisition to final reporting.

Finally, over the course of a five-year clinical trial it is possible that new data will have emerged to make the primary trial question less relevant, or that costs for key variables will have changed. In one recent study, 8,307 patients were randomly assigned to receive enoxaparin plus elastic stockings with graduated compression, or placebo plus elastic stockings with graduated compression [39]. Among all patients, there were similar 30-day mortality and major bleeding rates. Although important, there was no ability in the trial to detect actual VTE, which itself can have important clinical and economic impact aside from death. Also, although LMWH has historically had a higher drug acquisition cost than UFH, since the initiation of the PROTECT trial, the drug acquisition cost for LMWHs has diminished and UFH has increased [40]. This will be an important consideration for E-PROTECT in order to make the results contemporary and relevant.

In summary, administration of heparins is a commonly used strategy for VTE prevention. The PROTECT trial is important in determining the balance of effects, side effects and complications associated with LMWH and UFH thromboprophylaxis in medical-surgical ICU patients. The PROTECT trial leaves unanswered what consequence LWMH or UFH use has on the costs of care of patients with critical illness. E-PROTECT will complement the PROTECT trial with a pre-specified prospective comprehensive economic evaluation. Components of this study plan could be considered for other economic evaluations in critical care.

### Trial status

The PROTECT trial ClinicalTrials.gov number is NCT00182143. At the time of submission, the costing determination of E-PROTECT has not yet been completed and subsequent costs and analyses other than those reported here may be considered and reported, with justification.

## Electronic supplementary material

Additional file 1: **E-PROTECT: The economic evaluation of the PROTECT (Prophylaxis for ThromboEmbolism in Critical Care Trial) Study.** (DOC 182 KB)

## Abbreviations

ASA: Acetyl salicylic acid
CT: Computerized tomography
DVT: Deep vein thrombosis
ELISA: Enzyme-linked immunosorbent assay
HIT: Heparin-induced thrombocytopenia
HITT: Heparin induced thrombocytopenia with thrombosis
ICU: Intensive care unit
IU: International units
LWMH: Low molecular weight heparin
PE: Pulmonary embolism
PROTECT: Prophylaxis for ThromboEmbolism in Critical Care Trial
UFH: Unfractionated heparin
VTE: Venous thromboembolism.
