# Supplementary material for: Economic evaluation of the prophylaxis for thromboembolism in critical care trial (E-PROTECT): study protocol for a randomized controlled trial
Source: Trials. 2014 Dec 20;15:502. doi: 10.1186/1745-6215-15-502 (PMC4413997; doi:10.1186/1745-6215-15-502)
Supplement: Supplementary file 1 — Additional file 1: E-PROTECT: The economic evaluation of the PROTECT (Prophylaxis for ThromboEmbolism in Critical Care Trial) Study. (DOC 182 KB) [file 13063_2014_2417_MOESM1_ESM.doc]

**Additional file 1**

**E-PROTECT: The economic evaluation of the PROTECT (Prophylaxis for ThromboEmbolism in Critical Care Trial) Study**

**Operations Manual**

**Costing Methodology and Definitions**

Data Collection

***Clinical data*** on every patient will be collected as part of the PROTECT trial. Site coordinators have already participated in pilot trials, and undergone intensive training sessions to gain experience with the operations manual and case report forms of PROTECT. The Methods Centre at McMaster University will manage trial data and coordinate PROTECT case report form transmission. Case report form variables in PROTECT provided a robust accounting of patient characteristics at enrolment, length of stay, treatments and diagnostic testing received, outcomes, adverse events and lenghts of stay. We will obtain variable names from the Methods Centre at McMaster to associate with costs.

***Resource utilization.*** To determine the incremental cost of patients receiving LMWH compared to UFH, the resources consumed by patients in the PROTECT study, as defined by the eligibility criteria and actual enrolled patient characteristics will be collected. Enrolled patients are admitted to the intensive care unit in the hospital, administered LMWH or UFH as part of the PROTECT study, with daily follow-up, Doppler ultrasounds and study procedures, and follow-up for study outcome, complications, etc. In determining an incremental cost, only the resources that will differ between the two treatment options need to be identified. However, because the resources that will differ are uncertain, a prospective randomized trial and accompanying economic evaluation is being conducted. All important resources will be ascertained and analyzed. Once resources are identified, the amount of resources used and the unit costs of each resource used for a given patient need to be determined.

For purposes of an economic impact evaluation, resources must be translated into monetary values. Resource utilization variables associated with the direct medical costs of critically ill patients include: (1) Hospital and Critical Care; 2) Health Care Worker; 3) Medication; 4) Procedures; 5) Diagnostics and Laboratory; 6) Supplies and Equipment utilization; and 7) Complications. A comprehensive list of direct medical resource utilization elements associated with critically ill patients has been identified. As part of a pilot study involving six hospitals in Canada, the United States and Australia, we undertook to determine the feasibility of obtaining patient specific line-item costing. We discovered that in both privately funded and publically funded institutions, the variabiltiy around patient costing was substantial and that line-item costs were not routinely available; that many costs were “rolled up” into summary cost measures, and that this methodology would not allow for a linkage of costs and clincial events (the later measured as part of the PROTECT trial case report form). We thus have developed a cost gathering medology that captures hospital-specific line item costs, according to important variables that we anticipate will drive costs and possible cost-effectiveness, as determined by a systematic review of the literature of economic evaluation of VTE prevention for in-patients, our pilot study, the PROTECT case report form, and experts in the field of critical care the VTE.

***Quantification of Cost Variables***. Since this evaluation is a sub-study (“piggy-back”) of the larger PROTECT RCT, all resources associated with critical care patients receiving LMWH and UFH as part of the PROTECT study are identified and captured by the primary and co-investigators of the PROTECT trial, and at the completion of the study, will inform the resources used by patients. The PROTECT study case report form captures process of care, medication use, diagnostic testing, personnel use by days in ICU and hospital, complications such as bleeding, medication reactions, and clinical outcomes. Other events and recourses not captured as part of the case report form include most prominently, the actual costs associated with the events and recourses consumed by enrolled patients.

All direct medical resources for critically ill patients admitted to participating hospitals in PROTECT, will be identified during the prospective evaluation of the PROTECT study. Unit costs will be obtained from a number of source departments within participating hospitals and provincial or state sources. Costs will be collected in the units of the participating centre and converted and evaluated initially Canadian dollars, then to American dollars in the year of publication. Discounting will not be applied for for short-term (<1year) time-horizon events, but for modeled time-hoizons beyond 1 year, discounting at 3% will be applied.

***Notes on Unit Costs.*** A unit cost differs from a charge. Costs are the expenses incurred by the hospital for the service/procedure rendered. Charge is the amount that hospital requires drug companies/researchers to pay for a service/procedure to be conducted at their hospital. The charge consists of the cost of performing the service/procedure and a mark-up fee. Unit costs will be obtained by several methods.

1. Hospital budget

Ideally, all hospital costs should reflect expenses to the hospital budget. This information, if available would be obtained from hospital financial departments. However, in the vast majority of cases unit costs are not available for several reasons including:

1. Items are presented in bulk/mass quantity costs
2. Prices cannot be disclosed due to agreement with the supplier
3. Item costs are several years old
4. Costs are not available
5. Government reimbursement

Where hospital budget costs are not available, costs are obtained from government sources. In countries with public health care, the government is responsible for reimbursing health professionals, labs and hospitals for services rendered. Often a schedule of fees is produced by the government to outline the amount that can be reimbursed for each procedure or test. These schedules of fees were accessed to obtain unit costs. In some cases where schedule of benefits are restricted, the information was collected through a medical professional at a hospital associated with the PROTECT trial. In some jurisdictions, where there is a greater combination of public/private health care (e.g. US, Australia), the total private health care fee presented in the Medicare Benefits Schedule Book, or equivalent Government medical benefits schedule was used.

1. Charge to Cost Ratios

Where costs cannot be obtained, the amount that hospital charges for a procedure, either to patients or to investigators for clinical trials will be used where cost to charge ratios are available. We will use cost:charge ratios that relate to individual costs, as opposed to “rolled-up” ratios, as much as possible

**General Costing Procedures.**

The PROTECT site investigators list (maintained by the McMaster Methods Centre) is used to determine the initial contact individuals for costing information. An introductory e-mail is to be sent to all site investigators (and to the research coordinator if known) to inform them of E-PROTECT and requesting their assistance in providing E-PROTECT investigators with a contact with financial information from their site. If there is no response by the PROTECT site investigators, individuals were contacted 2 more times, with an attempt at telephone contact. If there is still no response, or the site investigators refused to participate, the site was excluded from analysis.

The general procedure for initiating the costing exercise at each hospital is as follows:

1. Contact site investigator and study team for most appropriate person to identify the following main costs.

2. Individual contacts provided by PROTECT site investigators will be contacted. These individuals will be informed of the study and hospital related costs were requested. In some cases PROTECT site investigators may prefer to contact the site themselves. The e-mail (below) will be sent to contacts.

3. For each cost item a person at the hospital most responsible for knowing/ determining the hospital-specific cost (e.g. radiology, blood bank, pharmacy, ICU human resources) will be contacted.

4. Each contact person will be asked if a hospital specific cost exists for each variable.

5. It will be further determined if the cost is an actual cost, or “charge”. If the item is a charge, a hospital line-item specific cost to charge ratio will be required.

6. If the cost is generalizable to a broader geography (health region laboratory cost, provincial physician reimbursement rate, etc.), then these costs will be obtained by the investigators and compared to the hospital specific costs. Significant discrepancies will be further interrogated to determine whether the difference is real, which best approximates actual cost (vs. charge). Notations will be made on the dataset and used for future decisions on which numbers to apply to eventual analyses. The list of study variables, definitions, and documentation examples for sources of variable values is below.

Sample Communication to Identified Individuals at E-PROTECT Sites

*Hello,*

*I am helping with the* ***economic evaluation of the PROTECT study****. We are in the process of gathering* ***costing data on key variables*** *and suspected drivers from sites in Canada, the US, Australia, Brazil and Saudi Arabia.*

*The goals will be* ***threefold:***

***(1)*** *first to describe* ***variability in costs*** *between sites, and among countries.*

***(2)*** *we hope to collect data on costing from most sites in PROTECT so that we will be able to* ***explore how variability in median*** ***costs reduces as more sites are added*** *- hopefully will be able to say something like "in a large multi-centre, international RCT of critically ill patients, we found that after gathering costing data from X sites, the variability in costs reduced to a sufficiently small amount to make further costing gathering unnecessary" (i.e. only need 12 sites of 30 in a large RCT, and x% from each participating country).*

***(3) site specific costing data is crucial*** *to the eventual E-PROTECT cost-efficacy study.*

*I have listed the key variables below that we are looking at right now and wonder if you might be able to put us on the right track of who to contact at your site .* ***We would like to include you in all three of these projects and publications****. Sometimes there is a costing person attached to ICU or a costing/charging department, sometimes we have found it necessary to track down someone in* ***radiology****,* ***pharmacy****,* ***ICU****,* ***lab*** *services, etc. - do you think you could help put us on the right track - with* ***names/emails*** *or by forwarding the request?*

*Note that we are NOT looking for any patient specific data, just generic costs for the specific items. Thanks so much!*

*Rob Fowler*

***E-PROTECT COST LIST***

**Pharmacy Costs** **- Just Tell us Who to Contact:**

*Unit cost for Dalteparin

*Unit cost for low dose heparin

Protamine Drug (per mg cost)

**Clinical Laboratory Costs** **- Just Tell us Who to Contact:**

*Anti-Xa level test

*Heparin induced thrombocytopenia assay (ELISA or a Serotonin-Release Assay)

*PTT/INR lab test

**General ICU and Wages Costs** **- Just Tell us Who to Contact:**

*Generic cost for a day of care in ICU

*Generic cost for a day of care on an in-patient ward

*Intensive Care unit physician cost/charge per day

*Nursing hourly rate for ICU

*Nursing hourly rate for ward

*Pharmacist hourly rate

Gastroscopy physician cost
Laparotomy physician costs

**Radiology Costs** **- Just Tell us Who to Contact:**

*Bilateral lower limb venous Doppler ultrasound to rule out DVT

*CT angiogram chest (pulmonary embolism protocol)

*Ventilation/Perfusion Scan of the lungs

*Chest X-ray

CT scan abdomen

CT scan pelvis

CT scan head

Angiography and Embolization of Bleeding Vessel

Vena cava filter insertion

**Blood Bank / Transfusion Services Costs** **- Just Tell us Who to Contact:**

***Transfusion of 1 unit of Red Blood Cells**

***Transfusion of 1 unit of Fresh Frozen Plasma**

**Definition of Variables, Source Documentation for Values**

**NOTE THAT DEFINITIONS MAY DIFFER IN ONE OR OTHER JURISDICTIONS. PLEASE USE THE DEFINITIONS AS A GUIDELINE.**

Drug costs

Unit cost to be paid by the hospital to the drug company as negotiated between the hospital and the drug company. The cost is usually found in the hospital drug formulary, or is known to the hospital pharmacy contact.

| **Variable** | **Definition** | **Units for costing determination (if applicable)** | **Source of definition (if applicable)** | **Captured in PROTECT CRF?** |
| --- | --- | --- | --- | --- |
| Dalteparin | Low molecular weight anticoagulant heparin by subcutaneous Dalteparin Sodium Injection | 5000 International Units/ 0.2mL in a prefilled syringe with safety needle device | E.g. hospital formulary pharmacy contact (name, date) | YES – by randomization allocation Form 5.1, and Form 4.2 |
| Unfractionated Heparin | Unfractionated heparin anticoagulant by subcutaneous injection | 5000 IU | E.g. hospital pharmacy contact (name, date) | YES – by randomization allocation Form 5.1, and Form 4.2 |
| Protamine drug | An unfractionated heparin intravenous reversal agent | 50 mg | E.g. hospital pharmacy contact (name, date) | Not specifically, but may be captured in free text or implied Forms 4.2, 9.1, 9.2, 12.2. May model this cost for this type of event (on IV heparin and major bleed) |
| Epinephrine or inotrope/vasopressor infusion costs | Epinephrine that is given continuously as a diluted liquid | Per microgram or milligram | E.g. hospital pharmacy contact (name, date) | Form 4.1 Inotropes/vasopressors; Form 7.1 – VTE Outcome events capture cardiopulmonary complications including arrest and hypotension |

Drug / Heparin Assay costs

Hospital cost for providing one assay, including materials costs and hospital overhead costs. If the laboratory providing the assay is external, the cost that the hospital is charged by the external laboratory will be used.

| **Variable** | **Definition** | **Units for costing determination (if applicable)** | **Source of definition (if applicable)** | **Captured in PROTECT CRF?** |
| --- | --- | --- | --- | --- |
| HIT assay- SRA | Serotonin release assay (SRA) is a laboratory test that confirms the diagnosis of a drug complication known as heparin-induced thrombocytopenia (HIT) | One assay | E.g. Bartholomew JR et al. 2005. Cleveland Clinic Journal of Medicine. 72, suppl 1, S31- S36 | Form 15.1 |
| HIT assay - screen | Hospital specific laboratory test that identifies the diagnosis of a drug complication known as heparin-induced thrombocytopenia (HIT) – operating characteristics are often less specific than SRA | One assay |  | Form 4.2 suspected HIT; Form 15.1 HIT testing |
| Heparin anti-Xa assay | An assay that determines the anticoagulant activity when patients are treated with low molecular weight heparin. | One assay |  | Form 4.2 anti-Xa level |

Physician costs

Cost that is reimbursed by the government authorities to the Physician for services rendered. Cost often found in a schedule of benefits.

| **Variable** | **Definition** | **Units for costing determination (if applicable)** | **Source of definition (if applicable)** | **Captured in PROTECT CRF?** |
| --- | --- | --- | --- | --- |
| Initial Critical Care physician fee | First day of Comprehensive Care rendered by “an Intensive Care physician who provides both Critical Care and Ventilation Support to patients in the Intensive Care Area. The service includes initial consultation and assessment and subsequent examinations, often including comprehensive critical care procedures such as endotracheal intubation, tracheal toilet, artificial ventilation and all necessary measures for respiratory support, emergency resuscitation, insertion of intravenous lines, cutdowns, intraosseous infusion, arterial and/or venous catheters pressure infusion set and pharmacological agents, insertion of C.V.P lines, defibrillation, cardioversion and usual resuscitative measures, insertion of urinary catheters and nasogastric intubation with or without anaesthesia, securing and interpretation of blood gases and laboratory tests, oximetry, transcutaneous blood gases, intracranial pressure monitoring interpretation and assessment when indicated (excluding insertion of I.C.P. measuring device).” | First episode/first day. | E.g. Ontario Ministry of Health and Long Term Care Schedule of Benefits: Physician Services. Similar definitions exist for other jurisdictions. | Form 3.1 ICU admission date |
| Daily Critical Care physician fee | Fee that is reimbursed to an Intensive Care physician for Comprehensive Care as defined above for a patient’s hospitalization from day 2 to 30 inclusive. | Daily rate. | E.g. Ontario Ministry of Health and Long Term Care Schedule of Benefits: Physician Services. Similar definitions exist for other jurisdictions. | Form 11.1 Dates between date of admission to ICU Form 3.1.3 OR date of entry into study Form 2.1 Randomization Date Form and date of discharge or death from ICU Form 11.1.5 |
| Initial Physician consultation fee (internal med) | “Admission assessment is a general assessment rendered to a patient on admission” to a long-term care institution: non-emergency in-patient services, including chronic care hospitals | First episode/first day. | E.g. Ontario Ministry of Health and Long Term Care Schedule of Benefits: Physician Services. Similar definitions exist for other jurisdictions. | Form 4.1 Surgical Consultation; Form 4.1 first episode of dialysis; possibly free text in Form 9.2 (bleeding outcome intervention/procedure); Form 11.1 (Period between Date of discharge from ICU and date of discharge from hospital with first day and subsequent days on non-ICU “ward”) |
| Initial physician fee (long-term care) | “Non-Emergency Long-Term Care In-Patient” “…services apply to patients in chronic care hospitals, convalescent hospitals, nursing homes, homes for the aged and designated chronic or convalescent care beds in hospitals other than patients in designated palliative care beds…”. “A Type 1 admission assessment is a general assessment rendered to a patient on admission.” | First episode/first day. | E.g. Ontario Ministry of Health and Long Term Care Schedule of Benefits: Physician Services. Similar definitions exist for other jurisdictions. | Not specifically, but Form 12.1 outlines transfer to another hospital |
| Daily physician fee (long-term care) | Fee that is reimbursed to a physician for services to a patient in chronic care or convalescent hospital during the “First 4 subsequent visits… per month”. “A subsequent visit is any routine assessment following the patient’s admission to a long-term care institution.” | Daily rate. | E.g. Ontario Ministry of Health and Long Term Care Schedule of Benefits: Physician Services. Similar definitions exist for other jurisdictions. | Not specifically, but Form 12.1 outlines transfer to another hospital |
| Physician discharge day fee | Fee that is reimbursed to the Most Responsible Physician at the day of discharge for rendering a subsequent visit. Completion of discharge summary by the physician within 48 hours of discharge, arrangement for follow-up of patient and prescription of discharge medications (if any) is required. | Last day | E.g. Ontario Ministry of Health and Long Term Care Schedule of Benefits: Physician Services. Similar definitions exist for other jurisdictions. | Form 11.1 - Date of discharge from hospital after already discharged from ward. |

Non-Physician Medical Personnel

Hourly wage that is paid by the hospital to the medical personnel for his/her health services. Wage does not include benefits, unless stated. Costs are often available through hospital financial department.

| **Variable** | **Definition** | **Units for costing determination (if applicable)** | **Source of definition (if applicable)** | **Captured in PROTECT CRF?** |
| --- | --- | --- | --- | --- |
| Respiratory therapy | Respiratory therapists assist physicians in the diagnosis, treatment and care of patients with respiratory and cardiopulmonary disorders. | Hourly wage | E.g. Service Canada- Labour Market Information- Job Descriptions. Similar definitions exist for other jurisdictions.  http://www.labourmarketinformation.ca/standard.asp?ppid=82&lcode=E&prov=1&gaid=1&occ=3214&job=&search_key=1&search_type=&employer_potential=&new_search= | Form 3.1 and Form 11.1 (Dates between ICU admission date *OR* first day in PROTECT and Discharge or death from ICU). Assume maximal period of respiratory therapy is while admitted to ICU. |
| Nursing | Provide direct nursing care to patients, deliver health education programs and provide consultative services regarding issues relevant to the practice of nursing. | Hourly wage | E.g. Service Canada- Labour Market Information- Job Descriptions. Similar definitions exist for other jurisdictions.  <http://www.labourmarketinformation.ca/standard.asp?ppid>=  82&lcode=E&prov=1&gaid=1&occ=3152&job=&search_  key=1&search_type=&employer_potential=&new_search= | Form 3.1 and Form 11.1 (Dates between ICU admission and discharge date or death from ICU or hospital *OR* first day in PROTECT and Discharge or death date from ICU or hospital) |
| Pharmacy time | Compound and dispense prescribed pharmaceuticals and provide consultative services to both clients and health care providers. | Hourly wage | E.g. Service Canada- Labour Market Information- Job Descriptions. Similar definitions exist for other jurisdictions.  <http://www.labourmarketinformation.ca/standard.asp?ppid>=  82&lcode=E&prov=1&gaid=1&occ=3131&job=&search_  key=1&search_type=&employer_potential=&new_search= | Form 3.1 and Form 11.1 (Dates between ICU admission and discharge date or death from ICU or hospital *OR* 1st day in PROTECT and Discharge or death date from ICU or hospital); Form 9.2 if bleeding outcome related med dispensed by pharmacy; Form 7.1 if VTE outcome required medication dispensed by pharmacy (e.g. other anticoagulant, thrombolytic therapy, etc.) |
| Physiotherapy time | Assess patients and plan and carry out individually designed treatment programs to maintain, improve or restore physical functioning, alleviate pain and prevent physical or respiratory dysfunction in patients. | Hourly wage | E.g. Service Canada- Labour Market Information- Job Descriptions. Similar definitions exist for other jurisdictions.  <http://www.labourmarketinformation.ca/standard.asp?ppid>=  82&lcode=E&prov=1&gaid=1&occ=3142&job=&search_  key=1&search_type=&employer_potential=&new_search= | Might estimate based on Form 3.1 and Form 11.1 (Dates between ICU admission and discharge date or death from ICU or hospital *OR* first day in PROTECT and Discharge or death date from ICU or hospital) |
| Social worker time | Help individuals, couples, families, groups, communities and organizations develop the skills and resources they need to enhance social functioning and provide counseling, therapy and referral to other supportive social services. | Hourly wage | E.g. Service Canada- Labour Market Information- Job Descriptions. Similar definitions exist for other jurisdictions.  http://www.labourmarketinformation.ca/standard.asp?ppid=82&lcode=E&prov=1&gaid=1&occ=4152&job=&search_key=1&search_type=&employer_potential=&new_search= | Might estimate based on Form 3.1 and Form 11.1 (Dates between ICU admission and discharge date or death from ICU or hospital *OR* first day in PROTECT and Discharge or death date from ICU or hospital). |
| Clerical time | Medical secretaries perform a variety of secretarial and administrative duties in doctor’s offices, hospitals, medical clinics and other medical settings. | Hourly wage | E.g. Service Canada- Labour Market Information- Job Descriptions. Similar definitions exist for other jurisdictions.  http://www.labourmarketinformation.ca/standard.asp?ppid=82&lcode=E&prov=1&gaid=1&occ=1243&job=&search_key=1&search_type=&employer_potential=&new_search= | Might estimate based on Form 3.1 and Form 11.1 (Dates between ICU admission and discharge date or death from ICU or hospital *OR* first day in PROTECT and Discharge or death date from ICU or hospital) |

Diagnostic Procedures

| **Variable** | **Definition** | **Units for costing determination (if applicable)** | **Source of definition (if applicable)** | **Captured in PROTECT CRF?** |
| --- | --- | --- | --- | --- |
| Unilateral lower limb ultrasound | Ultrasound imaging using Doppler technology for the lower extremities, focused upon the popliteal fossa and thigh, in order to diagnose deep vein thrombi (Imaging of one side of the leg through ultrasound). | 1 study. | E.g. Radiology Info (Web site developed and funded by: American College of Radiology (ACR) and Radiological Society of North America (RSNA)) | Form 4.3 Daily Data – whether U/S done; Form 6.1 date of U/S for DVT or PE |
| Bilateral | Imaging of both legs using the above description. | 1 study | Above | Form 4.3 Daily Data – whether U/S done; Form 6.1 date of U/S for DVT or PE |
| CT angiogram of the lungs | Computed tomography of the major blood vessels of the legs, to detect thrombosis. This procedure is conducted on a machine that spirals the camera around the patient. Identify pulmonary arteries in the lungs to rule out [pulmonary embolism](http://www.radiologyinfo.org/en/glossary/glossary1.cfm?term=pulmonary_embolism) and sometimes thrombosis (clots) in veins of the legs. | 1 study | E.g. Radiology Info (Web site developed and funded by: American College of Radiology (ACR) and Radiological Society of North America (RSNA))  CT Angiography (CTA):  http://www.radiologyinfo.org/en/info.cfm?pg=angioct | Form 6.2 Test for DVT or PE, point 4. |
| Ventilation and Perfusion scan | “A lung ventilation/perfusion scan, or “V/Q” scan, is a test that measures air and blood flow in the lungs… to help diagnose or rule out a pulmonary embolism.” | 1 study | E.g. U.S. Department of Health & Human Services- National Heart and Lung and Blood Institute Diseases and Conditions Index  Lung Ventilation/ Perfusion Scan:  http://www.nhlbi.nih.gov/health/dci/Diseases/lvq/lvq_whatis.html | Form 6.2 Test for DVT or PE, point 3 |
| Pulmonary angiogram | “An angiogram of the lung is a fluoroscopy to observe the flow in the blood vessels of the lung. Can also be used to find narrowing or a blockage in a blood vessel that slows or stops blood flow.” Is often a confirmatory or “gold standard” test when other tests (CT angiogram or V/Q Scans) are not diagnostic. | 1 study | E.g. BCHealth Guide  Angiogram of the Lung- Lung Angiogram, Pulmonary Angiogram:  <http://www.bchealthguide.org/kbase/topic/medtest/hw201194/descrip.htm> | Form 6.2 Test for DVT or PE, point 5 (Other tests) |
| Chest radiograph (portable) | The chest x-ray, performed portably at the patient’s bedside, in the ICU or ward, usually performed as one film, in the anterior-posterior position. | 1 study | E.g. Chest X-ray (Radiography):  http://www.radiologyinfo.org/en/info.cfm?pg=chestrad&bhcp=1 | Form 7.1 VTE Outcome point 4.1 and 4.2; Form 4.3, point 15 (PE diagnosed today) for event related CXR. Form 4B.1 point 4 for ward related PE suspicion. For CXR related to length of stay: Form 3.1 and Form 11.1 (Dates between ICU admission and discharge date or death from ICU or hospital *OR* first day in PROTECT and Discharge or death date from ICU or hospital).  Likely no impact in CEA. Of some interest in variability study. |
| CT abdomen | CT scan of the abdomen, in order to diagnose bleeding. Oral and/or intravenous contrast material may be used to better identify the source of bleeding. | 1 study | E.g. Computed Tomography (CT)- Abdomen and Pelvis:  http://www.radiologyinfo.org/en/info.cfm?PG=abdominct | Form 6.2 Test for DVT or PE, point 5c (Other tests) |
| CT Pelvis | See above in CT Abdomen | 1 study | As above | for DVT or PE, point 5c (Other tests) |
| CT head | CT scan as explained above of the head. | 1 study | As above | for DVT or PE, point 5c other (Other tests) |
| Electrocardiogram costs | An electrocardiogram, performed at the bedside of the patient, in the ICU or ward. | 1 study | E.g. BCHealth Guide  Electrocardiogram- ECG (Electrocardiogram), EKG (Electrocardiogram):  http://www.bchealthguide.org/kbase/topic/medtest/hw213248/descrip.htm | Form 7.1 VTE Outcome point 4.1 and 4.2 |
| Central IV access | Insertion of an intravenous catheter for administration of fluid or measurement of pressures, to a central vein (internal jugular, femoral, subclavian sites). | 1 procedure | E.g. Ministry of Health and Long Term Care Schedule of Benefits: Physician Services | Form 4.2 Baseline data point 9.1-9 |
| Peripheral IV access | Insertion of an intravenous catheter for administration of fluid or measurement of pressures, to a peripheral vein | 1 procedure | E.g. Ministry of Health and Long Term Care Schedule of Benefits: Physician Services | Assume change of peripheral IV every 3 days, may be of some importance in relation to length of stay, but will not be cost driver of CEA. Of mild importance in variability study. Form 3.1 and Form 11.1 (Dates between ICU admission and discharge date or death from ICU or hospital *OR* first day in PROTECT and Discharge or death date from ICU or hospital) |
| Chest X-ray (non portable) | See above in Chest radiograph (portable); this study can also but rarely be performed outside of the intensive care unit for stable patients. | 1 study | E.g. Chest X-ray (Radiography):  http://www.radiologyinfo.org/en/info.cfm?pg=chestrad&bhcp=1 | Form 4B.1 point 4 for ward related PE suspicion. For CXR related to length of stay: Form 3.1 and Form 11.1 (Dates between ICU discharge date and hospital discharge date).  Likely no impact in CEA. Of some interest in variability study. Likely all CXR portable in ICU and if clinically deteriorate on ward. |
| Vena cava filter- IVC Filter insertion physician costs | “A vena cava filter is an umbrella-shaped barrier device that is inserted into the large vein that returns blood to the heart from the abdomen and legs (inferior vena cava). This filter helps prevent blood clots that form in the deep veins of the lower limbs from travelling to the lungs and heart where they may block blood flow.” | 1 procedure | E.g. BCHealth Guide  Vena cava filter:  http://www.bchealthguide.org/kbase/glossary/ue417/ue4174/def.htm | Form 7.1 point 6 vena cava filter insertion |
| Vena cava filter – inferior vena cavogram physician costs | The process of obtaining images of the vena cava that sometimes occurs prior to insertion of a vena cava filter, and may occur even when a filter cannot be places. | 1 procedure | E.g. BCHealth Guide  Vena cava filter:  http://www.bchealthguide.org/kbase/glossary/ue417/ue4174/def.htm | Form 7.1 point 6 vena cava filter insertion but unable to insert filter, halting at cavogram. Unlikely to occur and very unlikely to be incrementally helpful beyond filter insertion. |
| Gastroscopy | “Gastroscopy is an examination of the esophagus, stomach, and duodenum (the first part of the small bowel) using a gastroscope with fiber optic visualization, performed usually in the ICU, occasionally in the endoscopy suite of a hospital.” | 1 procedure | E.g. Richmond Health Services- Part of the Vancouver Coastal Health Authority  Gastroscopy (Upper GI Endoscopy ) FAQs:  http://www.rhss.bc.ca/bins/content_page.asp?cid=106-133-138-152-674 | Form 9.1 point 1 gastrointesintal bleeding NG blood or hematemesis AND [point 2.A.1 life-threatening upper or lower GI bleed OR 2.A.3 drop in hemoglobin or Blood pressure or need for 2 or more RBC units] |
| Colonoscopy | A colonoscopy is an examination of a patient’s large intestine (colon and rectum), often to find areas of inflammation or bleeding. using a colonoscope with fiber optic visualization, performed usually in the ICU, occasionally in the endoscopy suite of a hospital.” | 1 procedure | E.g. BCHealth Guide  Colonoscopy:  http://www.bchealthguide.org/kbase/topic/medtest/hw209694/descrip.htm | Form 9.1 point 1.1 gastrointesintal bleeding melena or hematochezia AND [point 2.A.1 life-threatening upper or lower GI bleed OR 2.A.3 drop in hemoglobin or Blood pressure or need for 2 or more RBC units] |
| Angiography | An angiogram is used to examine the blood flow in arteries or veins to determine if there is blockage of the blood vessels. | 1 procedure | E.g. BCHealth Guide  Angiogram- Arteriography:  http://www.bchealthguide.org/kbase/topic/medtest/hw201112/descrip.htm | Form 9.1.1.1-1-4 Bleeding site AND 9.2.7.4 therapeutic procedure embolization |
| Bronchoscopy | A bronchoscopy examines the patient’s airway with a flexible fiberoptic bronchoscope, to determine if there may be an infection, obstruction due to secretions, a mass. | 1 procedure | E.g. BCHealth Guide  Bronchoscopy:  http://www.bchealthguide.org/kbase/topic/medtest/hw200474/descrip.htm | Form 9.1 point 1.2 respiratory AND [point 2.A.1 or 2.A.2 or 2.A.3 or 2.A.4 or 2.A.5 major bleeding] |
| Venography | A venography examines the patient’s veins with the help of an injectable dye |  | E.g. BCHealth Guide  Venogram  http://www.bchealthguide.org/kbase/topic/medtest/hw235506/descrip.htm | Form 8.1 Non-leg thrombosis point 2.2 Venogram |

Cost reimbursed by the governing authority to the primary physician for procedure that is rendered at a hospital. Costs often include a Professional component, and a Technical component.

The ***professional component*** consists of:

**A.** Providing clinical supervision, including approving, modifying and/or intervening in the

performance of the procedure where appropriate, and quality control of all elements of the

technical component of the procedure.

**B.** Performance of any clinical procedure associated with the diagnostic procedure which is not

separately billable (e.g. injections which are an integral part of the study) and of any fluoroscopy.

**C.** Where appropriate, post-procedure monitoring, including intervening except where this constitutes

a separately billable service.

**D.** Interpreting the results of the diagnostic procedure.

**E.** Providing premises for any aspect(s) of A and D that is(are) performed at a place other than the place in which the procedure is performed.

The ***technical component*** consists of:

**A.** Preparing the patient for the procedure.

**B.** Performing the diagnostic procedure or assisting in the performance of fluoroscopy.

**C.** Making arrangements for any appropriate follow-up care.

**D.** Providing records of the results of the procedure to the interpreting physician.

**E.** Discussion with, and providing information and advice to, the patient or patient’s

representative(s), whether by telephone or otherwise, on matters related to the service.

**F.** Preparing and transmitting a written, signed and dated interpretive report of the procedure to the referring physician.

**G.** Providing premises, equipment, supplies and personnel for all specific elements of the technical and professional components except for the premises for any aspect(s) of A and D of the professional component that is(are) not performed at the place in which the procedure is

performed.

Additional costs related to the procedure, such as equipment amortization, hospital overhead and material costs are not required, but if these costs are available please make a note of it.

Hospitalization costs:

| **Variable** | **Definition** | **Units for costing determination (if applicable)** | **Source of definition (if applicable)** | **Captured in PROTECT CRF?** |
| --- | --- | --- | --- | --- |
| Level III ICU | The definition for the ICU where the most intensive life-supporting care can be provided. In the Ontario context, ICU’s are designated Level III (all levels of cardiac and respiratory and other organ life support can be provided; nursing:patient ratio is usually 1:1 or 1:2); Level II (often patients can receive intravenous vasoactive medications, and occasionally have endotracheal intubation, but not mechanical ventilation; nursing ration is often 1:2–4); Level I ICU (can provide respiratory or cardiographic monitoring, possibly an arterial blood pressure or central venous catheter, but not generally intravenous vasoactive medications; nursing ratio often 1:3–4) | 1 day | E.g. critical care directorate web site of jurisdiction | Form 3.1 and Form 11.1 (Dates between ICU admission date *OR* first day in PROTECT and Discharge or death from ICU). |
| Medical ward bed | General in-patient ward bed in acute care hospital | 1 day | E.g. Ontario ministry of health and long-term care | Form 3.1 and Form 11.1.7 (Dates between ICU discharge date and date of Discharge or death date from hospital). |
| Hematology clinic visit fee | Clinic specializing in out-patient hematology related appointments. Costs encompass expenses associated with running the clinic (i.e. staff wages, equipment, use of hospital facilities). | 1 visit | E.g. Ontario ministry of health and long-term care | Form 11.1.1 yes PE or DVT in ICU OR 11.1.3 yes DVT or PE in hospital AND (11.1.6 yes discharged from hospital alive), then model hematology or ‘consultant’ visit at Q 3–6 months x 2 visits for post hospitalization modeling. |
| Neurology clinic visit fee | Clinic specializing in out-patient neurology related appointments. Costs encompass expenses associated with running the clinic (i.e. staff wages, equipment, use of hospital facilities). |  | E.g. Ontario ministry of health and long-term care | Form 9.1.1.5 yes intracranial bleed OR 11.1.3 yes DVT or PE in hospital AND 11.1.6 yes discharged from hospital alive, then model neurology ‘consultant’ visit at Q 3–6 months x 2 visits for post hospitalization modeling. |
| Pulmonary medicine clinic visit fee | Clinic specializing in out-patient pulmonary related appointments. Costs encompass expenses associated with running the clinic (i.e. staff wages, equipment, use of hospital facilities). |  | E.g. Ontario ministry of health and long-term care | Likely not contributory – can probably not incorporate in model. Costs will be same as medical subspecialist rates for hematology, neurology otherwise. |

Laboratory costs:

Cost reimbursed by the governing authority to the primary physician for laboratory test that is rendered at a hospital. The laboratory costs include:

1. Carrying out the laboratory procedure, including collecting specimens and processing of specimens
2. Interpreting and/or providing the results of the procedure, even where the interpreting physician is another physician
3. Discussion with and providing advice and information to the patient or patient’s representative(s) whether by telephone or otherwise, on matters related to service.
4. Providing premises, equipment, supplies and personnel for the specific elements and for any aspect(s) of the specific elements, of any service(s) that is(are) performed at the place in which the laboratory procedure is performed.

| **Variable** | **Definition** | **Units for costing determination (if applicable)** | **Source of definition (if applicable)** | **Captured in PROTECT CRF?** |
| --- | --- | --- | --- | --- |
| Arterial blood gas | “An arterial blood gas (ABG) test measures the acidity ([pH](javascript:popoffwindow('../../../glossary/stp13/stp1367/def.htm'))) and the levels of oxygen (PO2) and carbon dioxide (PCO2), bicarbonate (HCO3), and oxygen saturation in the blood.” | 1 test | E.g. BC Health Guide  Arterial Blood Gases:  <http://www.bchealthguide.org/kbase/topic/medtest/hw2343/descrip.htm>  Ministry of Health and Long Term Care Schedule of Benefits: Laboratory Services | Form 7.1.1 PE event OR model as daily event for patients on mechanical ventilation Form 4.1.4.2 OR Form 6.1.1 clinical suspicion of PE. Likely not influential in CEA, unless significant number of PE or cardiovascular events in one arm. Of some interest for variability study. |
| PTT/INR | A partial thromboplastin time (PTT) is a test that determines how long it takes for a patient’s blood to clot. Bleeding problems can be revealed from this test. Usually used to monitor anticoagulation effect of unfrationated intravenous heparin.  International normalized ratio (INR) is the standard method to report the time taken for blood to clot (prothrombin time, PT). Usually used to monitor anticoagulation effect of enteral warfarin. | 1 test | E.g. BC Health Guide  Partial Thromboplastin Time:  <http://www.bchealthguide.org/kbase/topic/medtest/hw203152/descrip.htm>  Anticoagulants for atrial fibrillation:  <http://www.bchealthguide.org/kbase/topic/detail/drug/hw160175/detail.htm> | Daily data Form 4.1.5 INR or PTT value or Form 4B.1.5 INR or PTT value. Likely not influential in CEA, unless significant number of PE or cardiovascular events requiring treatment doses of UFH in one arm. Of some interest for variability study. |
| Occult blood | “A fecal occult blood test finds blood in the stool by placing a small sample of stool on a chemically treated card, pad, or wipe. | 1 test | E.g. BC Health Guide  Fecal Occult Blood Test (FOBT):  http://www.bchealthguide.org/kbase/topic/medtest/hw227116/descrip.htm | Form 9.1.2.3 decrease in hemoglobin without other source (i.e. NOT 9.1.1.1-10 OR 9.1.2.A.1,2,4) . Unlikely helpful in CEA. Recommend to delete this cost from steering committee. |
| Complete blood count | A complete blood count gives important information about the kinds and numbers of cells in the blood, especially red blood cells, white blood cells and platelets. | 1 test | E.g. BCHealth Guide  Complete Blood Count (CBC): http://www.bchealthguide.org/kbase/topic/medtest/hw4260/descrip.htm | Daily data Form 4.1.5 hemoglobin value or Form 4B.1.5 hemoglobin value. Likely not influential in CEA, unless significant and differential number of bleeding events requiring treatment doses of UFH in one arm. Of some interest for variability study. |
| Electrolytes (Na, K, CO2) | An electrolyte panel is a blood test that measures the levels of electrolytes and carbon dioxide in your blood. | 1 test | E.g. BC Health Guide  Electrolyte Panel  http://www.bchealthguide.org/kbase/topic/special/tr6146/sec1.htm | Not in PROTECT CRF. Likely not influential in CEA, even if significant and differential length of stay. Of some interest for variability study. Could delete from costing exercise. |
| Creatinine | Creatinine tests measure the level of the waste product creatinine in your blood and urine. | 1 test | E.g. BC Health Guide  Creatinine and Creatinine Clearance  http://www.bchealthguide.org/kbase/topic/medtest/hw4322/descrip.htm | Daily data Form 4.1.5 Creatinine value or Form 4B.1.5 Creatinine value. Likely not influential in CEA, unless significant and differential number of renal failure events in one arm. Of some interest for variability study. |
| Blood Urea Nitrogen | A blood urea nitrogen test measures the amount of nitrogen in the blood. | 1 test | E.g. BC Health Guide  BUN (Blood Urea Nitrogen)  http://www.bchealthguide.org/kbase/topic/medtest/aa36271/descrip.htm | BUN specifically not captured in CRF. Daily data Form 4.1.5 Creatinine value or Form 4B.1.5 Creatinine value. Likely not influential in CEA, unless significant and differential number of renal failure events in one arm. Of some interest for variability study, but likely captured by Creatinine. Could delete from costing exercise. |

Transfusion and Blood Bank:

| **Transfusion Variable** | **Definition** | **Units for costing determination (if applicable)** | **Source of definition (if applicable)** | **Captured in PROTECT CRF?** |
| --- | --- | --- | --- | --- |
| Red Blood Cells (RBCs) | Costs include extraction, preparation, storage and shipment to the hospital, and eventually the laboratory procedures involved in administration to the patient, depending upon the hospital. | per unit | E.g. Bloody Easy – Ontario Transfusion Guide | Form 4.1.2.1 RBC # units on daily form.  Cross validation with Form 9.1.2.3 clinically important bleeding requiring >=2 units RBCs  Cross validation with Form 9.2.7.1 total transfusion units RBC |
| Fresh Frozen Plasma (FFP) | Costs include extraction, preparation, storage and shipment to the hospital, and eventually the laboratory procedures involved in administration to the patient, depending upon the hospital. | per unit | E.g. Bloody Easy – Ontario Transfusion Guide | Form 4.1.2.2 FFP # units on daily form  Cross validation with Form 9.2.7.1 total transfusion units FFP |
| Cryoprecipitate (cryo) | Costs include extraction, preparation, storage and shipment to the hospital, and eventually the laboratory procedures involved in administration to the patient, depending upon the hospital. | per unit | E.g. Bloody Easy – Ontario Transfusion Guide | Form 4.1.2.4 Cryo # units on daily form  Cross validation with Form 9.2.7.1 total transfusion units Cryo |
| Platelets (plts) | Costs include extraction, preparation, storage and shipment to the hospital, and eventually the laboratory procedures involved in administration to the patient, depending upon the hospital. | per “Pack” of platelets – defined variably at each hospital (often 4–5 units/hospital standard) | E.g. Bloody Easy – Ontario Transfusion Guide | Form 4.1.2.3 PLTS # units on daily form  Cross validation with Form 9.2.7.1 total transfusion units PLTS |
| Type and screen | A Type and screen test is the first step conducted in order to ensure “…serologic compatibility between the donor and the recipient…”. It consists of two tests, the “type” test and the “screen” test. The type test is done to reveal the type of ABO antigens that are on the red blood cells (RBC) of the patient. The screen test is conducted to “…determine whether the recipient has formed what are known as “unexpected” RBC antibodies.” “Unexpected” antibodies are “…antibodies to non-ABO antigens.” in patients who had many RBC transfusions. |  | E.g. Yazer MH. 2006. CMAJ. 174, 1, 29–32 and  Bloody Easy – Ontario Transfusion Guide | IF any number of units recording on Forms 4.1.2.1-4 daily data forms and no units transfused in prior 3 days.  OR cross validate with any recording of units on Form 9.2.7.1 of RBCs, PLTS, Cryo, FFP and no units transfused in prior 3 days |
| Cross Match | A crossmatch is a test conducted to determine if the RBCs of the donor and the plasma of the recipient are compatible. “It can be done serologically to ensure compatibility with both anti-ABO and non-ABO antibodies or by computer as a check on ABO compatibility.” |  | E.g. Yazer MH. 2006. CMAJ. 174, 1, 29–32  and  Bloody Easy – Ontario Transfusion Guide | IF any number of units recording on Forms 4.1.2.1-4 daily data forms and no units transfused in prior 3 days.  OR cross validate with any recording of units on Form 9.2.7.1 of RBCs, PLTS, Cryo, FFP and no units transfused in prior 3 days.  Likely combine with Type and Screen (i.e. just use one of these values) |
| Protamine | An unfractionated heparin intravenous reversal agent | Per unit | E.g. Bloody Easy – Ontario Transfusion Guide | Not captured in PROTECT CRF. Estimate if patient is on therapeutic IV UFH (Form 4.1.1.3) AND has a major bleed (Form 9.1.2.A.1-5)  Cross estimate if with Bleeding outcome Form 9.2.7.3 free text protamine. |
| DDAVP | Desmopressin is a drug that “… increases the amount of [clotting factor](javascript:popoffwindow('../../../../glossary/stc12/stc123740/def.htm')) VIII, which helps blood to clot. It is available in a nasal spray form that is often prescribed. Desmopressin can also be injected. Desmopressin is used for people with mild to moderate [von Willebrand's disease](javascript:popoffwindow('../../../../glossary/stv54/stv5464/def.htm')).” | Per unit | E.g. BCHealth Guide  E.g. Bloody Easy – Ontario Transfusion Guide  Desmopressin acetate for von Willebrand’s disease:  http://www.bchealthguide.org/kbase/topic/detail/drug/ue4013/detail.htm | Bleeding outcome Form 9.2.7.3 free text DDAVP. |
| Factor VIIa | Factor VII is a protein that is part of the chain of clotting factors which lead to the formation of a blood clot. | Per unit | E.g. National Hemophilia Foundation  Bloody Easy – Ontario Transfusion Guide  Factor VII Deficiency:  http://www.hemophilia.org/NHFWeb/MainPgs/MainNHF.aspx?menuid=187&contentid=50&rptname=bleeding | Bleeding outcome Form 9.2.7.3 free text Factor VII a or Activated Factor VII. |
| Thawing and allocation of plasma | Fresh frozen plasma must be thawed in a 37 degree water bath for 30 minutes before transfusion. | Per unit (200-250ml of plasma) | E.g. Transfusion Medicine Update – Institute for Transfusion Medicine, issue 1, 2004  http://www.itxm.org/tmu2004/issue2004-1.htm | Form 4.1.2.2 FFP # units on daily form  Cross validation with Form 9.2.7.1 total transfusion units FFP |
| Aprotinin | A proteinase inhibitor that inhibits plasmin – prevent/treat increased fibrinolysis during surgery. | Per unit | E.g. Bloody Easy – Ontario Transfusion Guide | Bleeding outcome Form 9.2.7.3 free text aprotinin. |
| Aminocaproic acid |  | Per unit | E.g. Bloody Easy – Ontario Transfusion Guide | Bleeding outcome Form 9.2.7.3 free text amicar or aminocaproic acid. |

Surgical procedure:

Surgical procedure costs would include the medical professionals, hospital bed cost for duration of procedure, equipment and administration.

| **Variable** | **Definition** | **Units for costing determination (if applicable)** | **Source of definition (if applicable)** | **Captured in PROTECT CRF?** |
| --- | --- | --- | --- | --- |
| Laparotomy- surgical fee | “Laparotomy is a surgical procedure that allows the surgeon to see and inspect the abdominal cavity for structural problems. This encompasses the surgeon fee; separate costs include the time for other operating room personnel, including nurses (often 2), an assistant physician, and overhead costs for the operating room (cleaning, power, etc.), captured variably at each hospital. | 1 procedure | E.g. BCHealth Guide  Surgery for chronic pelvic pain:  http://www.bchealthguide.org/kbase/topic/detail/surgical/tv2567/detail.htm | Form 9.1.2.5 bleeding requiring re-operation  Cross validate with Form 9.2.7 free text ‘surgery’ term for Bleeding outcome |
| Laparotomy- anaesthiology fee | See above in Laparotomy- surgical fee; the anesthesia component including pre-operative assessment of the patient, anesthesia during the procedure and post-operative care until the patient is discharged back to the care of the next responsible physician (e.g. the intensive care physician or surgeon) | 1 procedure | E.g. BCHealth Guide  Surgery for chronic pelvic pain:  http://www.bchealthguide.org/kbase/topic/detail/surgical/tv2567/detail.htm | Form 9.1.2.5 bleeding requiring re-operation  Cross validate with Form 9.2.7 free text ‘surgery’ term for Bleeding outcome |
| Laparotomy – assistant fee | See above in Laparotomy-surgical fee; Assistance at surgery include:   1. Preparing or supervising preparation of the patient for the procedure 2. Performing the procedure by any method, or assisting another physician in the performance of the procedure(s), assisting with carrying out of all recovery room procedures and transfer of the patient to the recovery room, and any ongoing monitoring and detention rendered during the immediate post-operative and recovery period, when indicated. 3. Making arrangements for any related assessments, procedures, or therapy (including obtaining any specimens from the patient) and/or interpreting results. 4. When medically indicated, monitoring the condition of the patient for post-procedure follow-up until the first post-operative visit. 5. Discussion with and providing any advice and information, including prescribing therapy to the patient or the patient’s representative(s), whether by telephone or otherwise, on matters related to the service 6. Providing premises, equipment, supplies and personnel for services for any aspect(s) of a, c, d and e that is (are) performed in a place other than the place in which the surgical procedure is performed. | 1 procedure | E.g. Ministry of Health and Long Term Care Schedule of Benefits: Physician Services | Form 9.1.2.5 bleeding requiring re-operation  Cross validate with Form 9.2.7 free text ‘surgery’ term for Bleeding outcome |
| Laparotomy – nursing fee | See above in Laparotomy-surgical fee; nurses assist surgery. | Per hour  For 1 procedure | E.g. as defined at hospital level and associated costs of nursing per hour or procedure in the operating room | Form 9.1.2.5 bleeding requiring re-operation  Cross validate with Form 9.2.7 free text ‘surgery’ term for Bleeding outcome |
| Nasal packing | Application of gauze or cotton packs to nasal chambers for the common purpose of controlling bleeding. | 1 procedure | E.g. Health A to Z  http://www.healthatoz.com/healthatoz/Atoz/common/standard/transform.jsp?requestURI=/healthatoz/Atoz/ency/nasal_packing.jsp | Form 9.1.2.B.2 nasal packing |
| Wound packing | Generally supplies for packing of a wound through the skin (gauze, 500 ml saline, sterile dressing tray). | 1 procedure | E.g. Health A to Z  http://www.healthatoz.com/healthatoz/Atoz/common/standard/transform.jsp?requestURI=/healthatoz/Atoz/ency/nasal_packing.jsp | Form 9.1.2.B.1 pressure bandage OR Form 9.1.2.B.3 other non-invasive intervention |

Cardiopulmonary arrest costs:

| **Variable** | **Definition** | **Units for costing determination (if applicable)** | **Source of definition (if applicable)** | **Captured in PROTECT CRF?** |
| --- | --- | --- | --- | --- |
| Cardiopulmonary arrest costs – physician cost | Service rendered when a physician provides resuscitation in emergency situations. The specific elements are those of an assessment, including immediate crisis-related examination, ongoing monitoring of the patient’s condition and the usual resuscitative procedures as required: defibrillation, cardioversion, cutdowns, intravenous lines, arterial and/or venous catheters, pressure infusion sets and pharmacological agents, urinary catheters, C.V.P. lines, blood gases, nasogastric intubation with or without anaesthesia, endotracheal intubation and tracheal toilet. | Per time of constant attendance | E.g. Ministry of Health and Long Term Care Schedule of Benefits: Diagnostic and Therapeutic procedures. | Form 7.1.4.5 Yes cardiopulmonary arrest |
| Cardiopulmonary arrest costs – nursing cost | Nurse assistance for resuscitation defined in “Cardiopulmonary arrest costs – physician cost”. | Per hour | E.g. as defined at hospital level and associated costs of nursing per hour or procedure in the operating room | Form 7.1.4.5 Yes cardiopulmonary arrest |
| Cardiopulmonary arrest costs – anaethesiologist cost | Anaethesiologist assistance for resuscitation defined in Cardiopulmonary arrest costs – physician cost | Per episode of care | E.g. Ministry of Health and Long Term Care Schedule of Benefits: Diagnostic and Therapeutic procedures. | Form 7.1.4.5 Yes cardiopulmonary arrest |
| Cardiopulmonary arrest costs – respiratory therapy cost | Non-anesthetist assistance for resuscitation defined in Cardiopulmonary arrest costs – RT cost | Per episode of care | Individual salary scale for RT involved in cardiac arrest | Form 7.1.4.5 Yes cardiopulmonary arrest |

Death:

This is variably considered in economic evaluations, but attempts to capture the cost to the hospital or health care system of a death, and incorporates such things as preparing the body for transportation to the morgue, preparation and resting there. We will capture this cost at centres where such costs are available or known.

| **Variable** | **Definition** | **Units for costing determination (if applicable)** | **Source of definition (if applicable)** | **Captured in PROTECT CRF?** |
| --- | --- | --- | --- | --- |
| Death | preparing the body for transportation to the morgue, preparation and resting etc., | 1 episode | E.g. Gould MK et al. Annals of Internal Medicine 1999. | Final Status Form 11.1.4 OR 11.1.6 was patient discharged from ICU or hospital alive NO |
